# Two new species of the *Liolaemus elongatus*-*kriegi* complex (Iguania, Liolaemidae) from Andean highlands of southern Chile

**DOI:** 10.3897/zookeys.500.8725

**Published:** 2015-04-27

**Authors:** Jaime Troncoso-Palacios, Hugo A. Díaz, Damien Esquerré, Felix A. Urra

**Affiliations:** 1 Programa de Fisiología y Biofísica, Instituto de Ciencias Biomédicas (ICBM), Facultad de Medicina, Universidad de Chile, Independencia 1027, Santiago, Chile; 2 Departamento de Ciencias Ecológicas, Facultad de Ciencias, Universidad de Chile, Las Palmeras 3425, Santiago, Chile; 3 Division of Evolution, Ecology and Genetics, Research School of Biology, The Australian National University, Canberra, ACT 0200, Australia; 4 Programa de Farmacología Molecular y Clínica, Instituto de Ciencias Biomédicas (ICBM), Facultad de Medicina, Universidad de Chile, Independencia 1027, Santiago, Chile

**Keywords:** *Liolaemus buergeri*, *Liolaemus kriegi*, new species, lizard, Laja Lagoon, Biobío

## Abstract

The *elongatus*-*kriegi* complex is one of the most diverse clades of the *Liolaemus* (*sensu stricto*) subgenus of lizards. There are currently 29 species recognized in this group distributed between Chile and Argentina. Based on molecular evidence, there seem to be five main clades nested within this complex: the *elongatus*, *leopardinus*, *kriegi*, *petrophilus* and *punmahuida* clades. *Liolaemus buergeri* and *Liolaemus kriegi*, both of the *kriegi* clade, were believed to inhabit the surroundings of the Laja Lagoon, in the Biobío Region of Chile. Moreover, this Chilean population of *Liolaemus kriegi* was recently recognized as an undescribed taxon called “*Liolaemus* sp. A” based on molecular phylogenetics. In this work, we studied these two populations of the Laja Lagoon and provided the morphological diagnosis to describe them as two new species: *Liolaemus scorialis* **sp. n.** and *Liolaemus zabalai* **sp. n.**, previously considered *Liolaemus buergeri* and “*Liolaemus kriegi*/*Liolaemus* sp. A” respectively. Additionally, we identified another population of *Liolaemus scorialis* in the vicinity of La Mula Lagoon in the Araucanía Region of Chile. *Liolaemus scorialis* differs from almost all of the species of the *elongatus*-*kriegi* complex by its considerably smaller size. Nevertheless, without molecular data we cannot assign it to any particular subclade. *Liolaemus zabalai* belongs to the *kriegi* clade based on published molecular phylogenies. Finally, we provide some natural history data on both species and we document for the first time the presence of *Liolaemus neuquensis* in Chile from a museum specimen from La Mula Lagoon.

## Introduction

*Liolaemus* is a diverse genus of South American lizards, with currently 245 species (Uetz and Hošek 2014) grouped into two subgenera: *Liolaemus* (*sensu stricto*) and *Eulaemus* (e.g. Laurent 1985, Schulte et al. 2000). Each of these subgenera has been divided into several groups based on phylogenetic relationships (Abdala 2007, Avila et al. 2006, Fontanella et al. 2012, Lobo 2005).

The *elongatus*-*kriegi* complex Cei (1979), is one of the most diverse groups of the *Liolaemus* (*sensu stricto*) subgenus with currently 29 species distributed in Chile and Argentina. In a phylogenetic study based on three mitochondrial genes, Morando et al. (2003) found that this complex is subdivided into three clades: *elongatus*, *kriegi* and *petrophilus*. Later, Avila et al. (2010a) based on one mitochondrial locus, added a fourth clade: the *punmahuida* clade. Finally, Esquerré et al. (2014) added a fifth clade comprising only Chilean endemic species: the *leopardinus* clade. An alternative classification has been proposed by Lobo (2005) and updated by Lobo et al. (2010b), based mainly on morphological and lifestyle traits, which classifies these species in three groups: *elongatus* (which includes the *capillitas* subgroup), *kriegi* and *leopardinus*, with a different arrangement compared with the molecular hypothesis (Table 1 and Table 2).

**Table 1.** Species of the *elongatus*-*kriegi* complex grouped by clades, based on mitochondrial molecular phylogenies. (1) Species included by Morando et al. (2003). (2) Species added by Avila et al. (2004). (3) Species added by Avila et al. (2010a). (4) Species added by Avila et al. (2012). (5) Species added fide Esquerré et al. (2014). *Liolaemus thermarum* is included in the *elongatus* clade by Avila et al. (2010a) but omitted by Avila et al. (2012).

*elongatus* clade	*kriegi* clade	*leopardinus* clade	*petrophilus* clade	*punmahuida* clade
*Liolaemus antumalguen* (3)	*Liolaemus buergeri* (1)	*Liolaemus frassinettii* (5)	*Liolaemus austromendocinus* (1)	*Liolaemus flavipiceus* (3)
*Liolaemus burmeisteri* (4)	*Liolaemus kriegi* (1)	*Liolaemus leopardinus* (5)	*Liolaemus capillitas* (1)	*Liolaemus punmahuida* (3)
*Liolaemus elongatus* (1)		*Liolaemus ramonensis* (5)	*Liolaemus dicktracyi* (2)	
*Liolaemus smaug* (4)		*Liolaemus ubaghsi* (5)	*Liolaemus gununakuna* (2)	
*Liolaemus thermarum* (3)		*Liolaemus valdesianus* (5)	*Liolaemus parvus* (3)	
			*Liolaemus petrophilus* (1)	
			*Liolaemus talampaya* (2)	
			*Liolaemus tulkas* (3)	
			*Liolaemus umbrifer* (2)	

**Table 2.** Species of the *elongatus*-*kriegi* complex by groups, based on morphological, skeletal and lifestyle traits phylogeny according to (1) Lobo (2005), (2) updated by Lobo et al. (2010b) and (3) fide Esquerré et al. (2013). The *capillitas* subgroup is nested into *elongatus* group (Lobo et al. 2010b).

*capillitas* subgroup	*elongatus* group	*kriegi* group	*leopardinus* group
*Liolaemus capillitas* (1)	*Liolaemus austromendocinus* (2)	*Liolaemus buergeri* (1)	*Liolaemus frassinettii* (2)
*Liolaemus dicktracyi* (1)	*Liolaemus carlosgarini* (3)	*Liolaemus cristiani* (1)	*Liolaemus leopardinus* (1)
*Liolaemus heliodermis* (1)	*Liolaemus elongatus* (1)	*Liolaemus kriegi* (1)	*Liolaemus ramonensis* (1)
*Liolaemus talampaya* (2)	*Liolaemus flavipiceus* (2)		*Liolaemus valdesianus* (1)
*Liolaemus tulkas* (2)	*Liolaemus gununakuna* (2)		
*Liolaemus umbrifer* (1)	*Liolaemus parvus* (2)		
	*Liolaemus petrophilus* (2)		
	*Liolaemus punmahuida* (2)		
	*Liolaemus thermarum* (2)		
	*Liolaemus tregenzai* (2)		

Currently, the *elongatus*-*kriegi* complex (Avila et al. 2012, Esquerré et al. 2014, Morando et al. 2003) or *elongatus*, *kriegi* and *leopardinus* groups (Lobo 2005, Lobo et al. 2010b) includes the following species: *Liolaemus antumalguen* Avila et al., 2010, *Liolaemus austromendocinus* Cei, 1974, *Liolaemus buergeri* Werner 1907, *Liolaemus burmeisteri* Avila et al. 2012, *Liolaemus carlosgarini* Esquerré et al. 2013, *Liolaemus capillitas* Hulse, 1979, *Liolaemus choique* Abdala et al. 2010, *Liolaemus cristiani* Núñez et al. 1991, *Liolaemus dicktracyi* Espinoza & Lobo, 2003, *Liolaemus elongatus* Koslowsky, 1896, *Liolaemus flavipiceus* Cei & Videla, 2003, *Liolaemus frassinettii* Núñez, 2007, *Liolaemus gununakuna* Avila et al. 2004, *Liolaemus heliodermis* Espinoza et al. 2000, *Liolaemus kriegi* Müller & Hellmich, 1939, *Liolaemus leopardinus* Müller & Hellmich, 1932, *Liolaemus parvus* Quinteros et al. 2008, *Liolaemus petrophilus* Donoso-Barros & Cei, 1971, *Liolaemus punmahuida* Avila et al. 2003, *Liolaemus ramonensis* Müller & Hellmich, 1932, *Liolaemus shitan* Abdala et al. 2010, *Liolaemus smaug* Abdala et al. 2010, *Liolaemus talampaya* Avila et al. 2004, *Liolaemus thermarum* Videla & Cei, 1996, *Liolaemus tregenzai* Pincheira-Donoso & Scolaro, 2007, *Liolaemus tulkas* Quinteros et al. 2008, *Liolaemus ubaghsi* Esquerré et al. 2014, *Liolaemus umbrifer* Espinoza & Lobo, 2003 and *Liolaemus valdesianus* Hellmich, 1950.

*Liolaemus buergeri*, of the *kriegi* clade, was described from El Planchón Volcano, Maule Region, Chile (Werner 1907). This species has been traditionally believed to be widely distributed in Chile and Argentina (Cei 1986, Pincheira-Donoso 2001). However, its current wide distribution is in part due to cases of misidentification and a lumping of cryptic species (Medina et al. 2013). Donoso-Barros (1970) extended the southern distribution of *Liolaemus buergeri* to the Andes of Talca, Maule Region, Chile (50 km S from El Planchón Volcano). Later, Pincheira-Donoso (2001) extended the Chilean southern distribution of *Liolaemus buergeri* to the Batea-Mahuida Volcano (Araucanía Region, 240 km S from El Planchón Volcano) and pointed out that he also examined three specimens from the Laja Lagoon (Biobío Region, Chile, 150 km S from El Planchón Volcano); but Pincheira-Donoso and Núñez (2005) indicated that the specimens from Batea-Mahuida Volcano indeed correspond to *Liolaemus elongatus*, whereas the status of “*Liolaemus buergeri*” from the Laja Lagoon in Chile remains uncertain. In regards to Argentina, Cei (1986) stated that this species occurs in Mendoza and Neuquén Provinces, but Morando et al. (2003) and Medina et al. (2013), based on genetic and morphological evidence, respectively, indicated that several Argentinean populations attributed to *Liolaemus buergeri* correspond to at least three undescribed species.

*Liolaemus kriegi*, also of the *kriegi* clade, was described from Estancia El Cóndor, Río Negro Province, Argentina (Müller and Hellmich 1939a). Later, Donoso-Barros (1966) extended its northern distribution to the Cordillera de Curicó, Maule Region, Chile, 650 km N of Estancia El Cóndor; and to the Laja Lagoon, Biobío Region, Chile, 400 km N of Estancia El Cóndor (Donoso-Barros 1974). Morando et al. (2003), based on mitochondrial genes, found three candidate species related to *Liolaemus kriegi*, all from Argentina and previously assigned to *Liolaemus buergeri*: *Liolaemus* sp. A (from Caviahue, Neuquén Province), *Liolaemus* sp. B (from Ranquil Norte, Neuquén Province) and *Liolaemus* sp. C (from Laguna Los Barros, Neuquén Province). Medina et al. (2013), in a morphological analysis of these populations, corroborated the status of candidate species of these *Liolaemus* sp., adding new localities for *Liolaemus* sp. A, including samples from the Laja Lagoon (Chile) which corresponds to the species previously identified as *Liolaemus kriegi* by Donoso-Barros (1974). Also, Medina et al. (2013) found another candidate species from Argentina (*Liolaemus* sp. D), previously identified as *Liolaemus buergeri* by Morando et al. (2003). Recently, Medina et al. (2014) in a new phylogenetic study based on mitochondrial and nuclear genes, corroborate the previous studies and provide strong evidence for *Liolaemus* sp. A as a candidate species, also based on samples from Chile (Laja Lagoon) and Argentina (several localities of Neuquén Province).

Here, we studied the taxonomic status of the southernmost currently-recognized Chilean population of “*Liolaemus buergeri*”, from the vicinity of the Laja Lagoon, Biobío Region; and of “*Liolaemus kriegi*/*Liolaemus* sp. A” from the same locality. This population of “*Liolaemus buergeri*” is described as a new species which differs greatly from *Liolaemus buergeri* and almost all species of the *elongatus*-*kriegi* complex by its small snout-vent length (less than 70.0 mm). Additionally, specimens of this new species are recorded from La Mula Lagoon, Araucanía Region, Chile. For “*Liolaemus kriegi*/*Liolaemus* sp. A”, we provide a full description and diagnosis of this new species belonging to the *kriegi* clade.

## Materials and methods

We examined specimens of almost all Chilean species currently considered as belonging to the *Liolaemus elongatus*-*kriegi* complex. The morphological characters were examined according to Etheridge (1995), Lobo (2005), Abdala et al. (2010) and Avila et al. (2010a, 2012). Body measurements were taken with a digital vernier caliper (0.02 mm precision). Measurements are provided as mean ± standard deviation (x ± SD). The Mann–Whitney U test was used to compare the new species and some related species. Scales were observed with different magnifying lenses and scalation and measurements were recorded on the right side of the specimen, unless otherwise indicated. Dorsal scales were counted between the occiput and the level of the anterior border of the hind limbs. Ventral scales were counted from mental scale to the anterior margin of cloacal opening. Stomach and intestinal contents were observed under a binocular microscope for one specimen of each new species. The specimens examined are listed in Appendix 1. Data for Argentinean species were taken from the literature. *Liolaemus ceii* is not accepted as valid species in this work (see discussion). Museum codes are as follow: MRC (Museo Regional de Historia Natural, Concepción), MZUC (Museo de Zoología, Universidad de Concepción) and SSUC (Colección de Flora y Fauna Patricio Sánchez Reyes, Pontificia Universidad Católica de Chile).

## Results

### Liolaemus scorialis sp. n.

Taxon classificationAnimaliaSquamataLiolaemidae

http://zoobank.org/35B1E4BC-4EA1-4FEF-B025-B93D5C5A9CB9

Fig. 1

Liolaemus buergeri (in part?), Pincheira-Donoso, 2001. Not. Mens. Mus. Nac. Hist. Nat., Chile, 346: 8.
Liolaemus buergeri (in part?), Pincheira-Donoso & Núñez, 2005. Pub. Oc. Mus. Nac. Hist. Nat., Chile, 59: 285.

#### Holotype.

**Figure 1.**
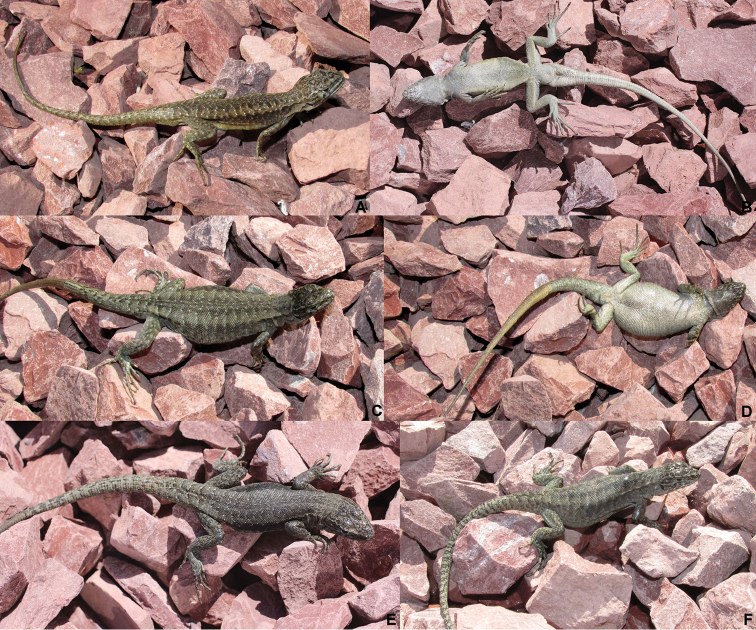
*Liolaemus scorialis* sp. n. **A, B** Holotype, male **C, D** Paratype, female **E** Paratype, male **F** Paratype, female. All from the type locality, 7 km NW of the summit of the Antuco Volcano, near the Laja Lagoon, Biobío Region, Chile.

SSUC Re 617 (Fig. 1). Male collected 7 km NW of the summit of the Antuco Volcano, near the Laja Lagoon, Biobío Region, Chile (37°21'S – 71°23'W, 1450 m). Collected by J. Troncoso-Palacios, F. Urra and H. Díaz. 08/01/2014.

#### Paratypes.

SSUC Re 615–16 two males and 612–614 three females (Figs 1 and 3). The same data as the holotype. MRC 675, 677, 680, 682. Four males. La Mula Lagoon (37°53'S – 71°22'W), Ralco National Reserve. Unknown coll. 01/12/2001.

#### Etymology.

The species name refers to the habitat, which is composed of accumulations of igneous rocks from the Antuco Volcano, called “scoria” from the Greek “skoria”. We propose the common name “Slag Lizard” in English and “Lagarto del escorial” in Spanish.

#### Diagnosis.

*Liolaemus scorialis* belongs to the *elongatus*-*kriegi* complex, but its specific assignation to a particular subclade is currently unknown since we have no molecular data for this new species, and molecular and morphological phylogenies for the *elongatus*-*kriegi* complex disagree in the arrangement of this complex subgroups (see discussion).

**Figure 2.**
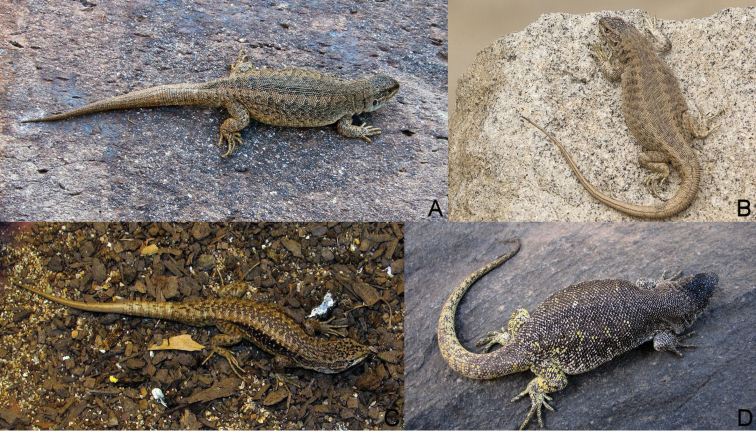
Chilean species of the *elongatus*-*kriegi* complex that live near the distribution of *Liolaemus scorialis* sp. n. and *Liolaemus zabalai* sp. n. **A** *Liolaemus buergeri* from El Planchón (type locality, photos by J. Troncoso-Palacios) **B** *Liolaemus buergeri* from Altos de Lircay (photos by R. Díaz) **C** *Liolaemus carlosgarini* from the road to the Maule Lagoon (type locality, photos by J. Troncoso-Palacios) **D** *Liolaemus flavipiceus* from the Maule Lagoon (photos by C. Garín).

Below a wide diagnosis is provided on aspect of all species of the complex. *Liolaemus scorialis* differs from almost all species of the *elongatus*-*kriegi* complex by its size (maximum SVL = 69.9 mm), smaller than *Liolaemus antumalguen* (Table 3), *Liolaemus austromendocinus* (max. SVL = 103.0 mm, Espinoza et al. 2000), *Liolaemus buergeri* (Table 3, Fig. 2), *Liolaemus burmeisteri* (Table 3), *Liolaemus capillitas* (max. SVL = 93.0 mm, Espinoza et al. 2000), *Liolaemus choique* (Table 3), *Liolaemus dicktracyi* (max. SVL = 91.0 mm, Espinoza and Lobo 2003), *Liolaemus elongatus* (max. SVL = 94.7 mm, Avila et al. 2012), *Liolaemus flavipiceus* (Table 3, Fig. 2), *Liolaemus frassinettii* (max. SVL = 91.1 mm), *Liolaemus gununakuna* (max. SVL = 97.5 mm, Avila et al. 2004), *Liolaemus kriegi* (max. SVL = 101.0 mm; Avila et al. 2003), *Liolaemus leopardinus* (max. SVL = 98.2 mm), *Liolaemus petrophilus* (max. SVL = 100.0 mm; Espinoza et al. 2000), *Liolaemus punmahuida* (Table 3), *Liolaemus ramonensis* (max. SVL = 94.9 mm), *Liolaemus shitan* (max. SVL = 98.3 mm, Abdala et al. 2010), *Liolaemus talampaya* (max. SVL = 85.5 mm, Avila et al. 2004), *Liolaemus thermarum* (max. SVL = 85.0 mm, Videla and Cei 1996), *Liolaemus tregenzai* (Table 3), *Liolaemus ubaghsi* (max. SVL = 89.6 mm), *Liolaemus umbrifer* (max. SVL = 89.0 mm, Espinoza and Lobo 2003), *Liolaemus valdesianus* (max. SVL = 93.4 mm) and “*Liolaemus kriegi*/*Liolaemus* sp. A” (max. SVL = 92.0 mm, described below).

**Table 3.** Scalation and morphological characteristics for the species of the *Liolaemus elongatus*-*kriegi* complex occurring near *Liolaemus scorialis* sp. n. and *Liolaemus zabalai* sp. n. distribution. Juvenile specimens examined are excluded. Source of data for not examined species are: *Liolaemus antumalguen* (Avila et al. 2010a), *Liolaemus burmeisteri* (Avila et al. 2012), *Liolaemus choique* (Abdala et al. 2010), *Liolaemus punmahuida* (Avila et al. 2004) and *Liolaemus tregenzai* (Pincheira-Donoso and Scolaro 2007). (*) Medina et al. (2013). M = males; F = females.

	*Liolaemus antumalguen*	*Liolaemus buergeri* (M = 5, F = 9)	*Liolaemus burmeisteri*	*Liolaemus carlosgarini* (M = 6, F = 11)	*Liolaemus choique*	*Liolaemus flavipiceus* (M = 5, F = 10)	*Liolaemus punmahuida*	*Liolaemus scorialis* sp. n. (M = 7, F = 3)	*Liolaemus tregenzai*	*Liolaemus zabalai* sp. n. (M = 3, F = 5)
Maximum SVL (mm)	107.8	96.2	85.2	68.8	90.7	95.8	96.0	69.9	90.2	92.0
Midbody scales	72–82	80–100	70–81	80–95	74–88	68–77	67–81	76–90	71–85	90–104
Dorsal scales	70–78	78–91	76–85	68–82	65–81	60–71	70–78	74–81	-	86–96
Ventral scales	105–118	111–125	99–110	112–124	118–135	93–105	-	115–131	-	116–122
Sexual dichromatism	Absent	Absent	Absent	Absent	Absent	Absent	Absent	Slight	Present	Slight
Cloacal region color (males)	Yellowish in some specimens but usually black	Yellowish	Yellowish	Yellowish	Yellowish	Reddish or yellowish in some specimens but usually black	Reddish or yellowish	Yellowish	-	Yellowish
Tail pattern	Absent	Vertebral line with diffuse rings in the tail base	Weak rings	Rigns (marked or weak)	Absent	Absent or weak rings	Absent	Rings	Rings	Rings
Precloacal pores on males	3–4	3–4	0–5	0–3	3–4	0	0	3–4	0	3–4 (3–5*)

*Liolaemus scorialis* has probably been previously confused with *Liolaemus buergeri* (see discussion), but in addition to the size difference, *Liolaemus scorialis* differs from *Liolaemus buergeri* because the latter has a vertebral stripe on the tail, whereas the tail is ringed in *Liolaemus scorialis*. Moreover, *Liolaemus buergeri* has more midbody scales (x = 89.4 ± 5.5, n = 14) than *Liolaemus scorialis* (x = 82.0 ± 4.7, n = 10) (Mann–Whitney U = 20.5, P < 0.01, DF = 21) and more dorsal scales (x = 84.1 ± 4.4) than *Liolaemus scorialis* (x = 76.5 ± 4.3) (Mann–Whitney U = 15.0, P < 0.01, DF = 21); but *Liolaemus buergeri* has fewer ventral scales (x = 118.7 ± 4.7) than *Liolaemus scorialis* (x = 124.0 ± 6.0) (Mann–Whitney U = 36.0, P = 0.05, DF = 21).

*Liolaemus scorialis* is syntopic with “*Liolaemus kriegi*/*Liolaemus* sp. A”, but in addition to the size difference, the latter has more midbody scales (x = 94.3 ± 4.8, n = 8) than it (Mann–Whitney U = 1.5, P < 0.01, DF = 16). Moreover, the dorsal scale count range of *Liolaemus scorialis* does not overlap with the range of “*Liolaemus kriegi*/*Liolaemus* sp. A” (Table 3). There is a black lateral band running from the tip of snout to the groin in “*Liolaemus kriegi*/*Liolaemus* sp. A”, whereas in *Liolaemus scorialis* there is a dark brown lateral band running from the shoulder to the groin.

*Liolaemus scorialis* differs from similar size species of the *elongatus*-*kriegi* complex as follows. *Liolaemus scorialis* differs from *Liolaemus cristiani* because the males of the latter lack precloacal pores and have reddish ventral coloration, whereas males of *Liolaemus scorialis* have 3–4 precloacal pores and no reddish ventral coloration.

*Liolaemus scorialis* differs from *Liolaemus heliodermis*, because the males of the latter have a black head and sulfur-yellow dorsum (Espinoza et al. 2000), an unique feature in the Liolaemus subgenus. Moreover, *Liolaemus heliodermis* has 62–69 midbody scales (Espinoza et al. 2000), whereas *Liolaemus scorialis* has 76–90.

*Liolaemus scorialis* differs from *Liolaemus parvus*, because the latter has 60–77 midbody scales and 96–113 ventral scales (Quinteros et al. 2008), whereas *Liolaemus scorialis* has 76–90 midbody scales and 115–131 ventral scales. *Liolaemus scorialis* has a ringed tail, whereas *Liolaemus parvus* has weak or absent rings on the tail (Quinteros et al. 2008).

*Liolaemus scorialis* differs from *Liolaemus smaug*, because the latter has marked sexual dichromatism with white spots dispersed on the dorsum of males and absent in females (Abdala et al. 2010), whereas both males and females of *Liolaemus scorialis* have white spots on the dorsum. *Liolaemus scorialis* has ringed tail, whereas *Liolaemus smaug* has weak or no rings on the tail (Abdala et al. 2010). Males of *Liolaemus smaug* have bright golden yellow dorsal color, a trait absent in *Liolaemus scorialis*.

*Liolaemus scorialis* differs from *Liolaemus tulkas*, because the males of the latter have 0–1 precloacal pores (Quinteros et al. 2008), whereas males of *Liolaemus scorialis* have 3–4 precloacal pores. Moreover, *Liolaemus tulkas* has 63–68 midbody scales (Quinteros et al. 2008), whereas *Liolaemus scorialis* has 76–90.

**Figure 3.**
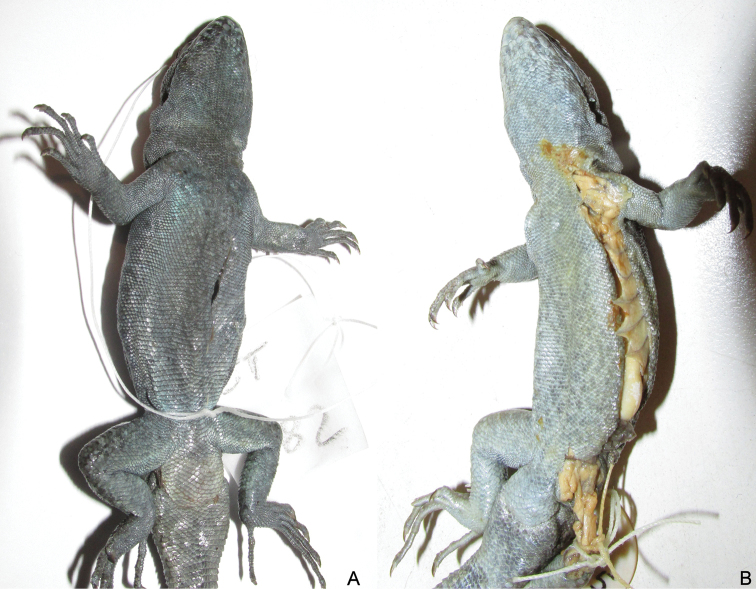
Comparison of the ventral color pattern. **A** *Liolaemus scorialis* sp. n. from type locality, with immaculate gray ventral color **B** *Liolaemus carlosgarini* with light gray ventral color and dark inconspicuous spots dispersed.

*Liolaemus scorialis* differs from *Liolaemus carlosgarini* (Fig. 2), because the males of the latter have 0–3 precloacal pores (present in 50% of the males, these are small and underdeveloped), whereas males of *Liolaemus scorialis* have 3–4 well developed precloacal pores. *Liolaemus scorialis* has more ventral scales (x = 124 ± 6.0, n = 10) than *Liolaemus carlosgarini* (x = 115 ± 4.0, n = 17) (Mann–Whitney U = 11.0, P = 0.01, DF = 25). Moreover, *Liolaemus scorialis* has brown dorsal color and immaculate gray ventral color, whereas *Liolaemus carlosgarini* has light brown-yellowish dorsal color and whitish ventral color with dark inconspicuous spots on the gular region and belly (Figs 2 and 3).

#### Description of the holotype.

Adult male. SVL 62.3 mm. Tail length 101.5 mm (not autotomized). Axilla-groin length 26.3 mm. Head length (from the posterior border of the auditory meatus to the tip of the snout) 16.4 mm. Head width (distance between the two ear openings) 11.4 mm. Head height (at the level of ear openings) 6.9 mm. Forelimb length 21.1 mm. Hindlimb length 39.7 mm. Foot length 18.9 mm. Rostral scale wider (2.5 mm) than high (1.0 mm). Two postrostrals. Four internasals. Hexagonal interparietal scale, with a central, small, and whitish spot marking the position of the parietal eye. Interparietal smaller than parietals, surrounded by six scales; nine scales between the interparietal and rostral (both excluded); 15 scales between occiput and rostral; orbital semicircle complete on the right side, formed by 13 scales, incomplete on the left side; 6-5 supraoculars (left-right); six superciliary scales. Frontal area is divided into six scales (two posterior, one in the center and three anterior); 2 scales between nasal and canthal; preocular separated from the lorilabials by one loreal scale; nasal in contact with the rostral, surrounded by seven scales. There is one row of lorilabials between the supralabials and the subocular. Seven supralabials, the fifth is curved upward without contacting the subocular. Four infralabial scales. Mental scale pentagonal, in contact with four scales; four pairs of postmental shields, the second is separated by two scales. Temporal scales are subimbricated and slightly keeled. There are ten temporal scales between the level of superciliary scales and the rictal level. Three projected scales on the anterior edge of the ear, which are small and do not cover the auditory meatus; auricular scale is wide and is restricted to the upper third of the meatus. Forty gulars between the auditory meatuses. Well developed “Y” shaped lateral neck fold and dorsolateral fold slightly developed. Antehumeral fold present. Midbody scales 88. Dorsal scales of the vertebral zone lanceolate, imbricate, keeled and without mucrons. Dorsal scales of the paravertebral fields more rounded, subimbricate, with more poorly developed keel, without mucrons and with interstitial granules between them. Dorsal scales of the vertebral zone are larger than the ventral scales. Dorsal scales of the paravertebral fields are similar in size to the ventral scales. Dorsal scales 81. Ventral scales are rhomboidal to rounded, smooth, imbricate, and without interstitial granules. Ventral scales 131. There are four precloacal pores. The suprafemoral scales are rhomboidal to rounded, imbricate, and smooth or slightly keeled. Infrafemoral scales are rounded, smooth, and imbricate. Supra-antebrachials scales are rhomboidal to rounded, imbricate, and slightly keeled or smooth. Infra-antebrachials are rounded to rhomboidal, subimbricate with few interstitial granules, and smooth. The dorsal scales of the tail are rhomboidal, imbricate, keeled and some with mucrons. The ventral scales of the tail vary from rhomboidal to triangular, and are imbricate and smooth. Lamellae of the fingers: I: 10, II: 17, III: 21, IV: 23 and V: 13. Lamellae of the toes: I: 13, II: 18, III: 22, VI: 29 and V: 20.

#### Color of the holotype in life.

Light brown head, with dark brown lines: a “Ω” shaped line between nasal scales and supraocular area, two short stripes on the posterior supraocular areas, an incomplete “O” shaped dark brown line surrounding the interparietal scale, six dark brown short lines on the occipital area. The temporal area is brown with two dark brown horizontal stripes; the ocular area and the cheeks are light gray. Subocular area is gray with two dark brown vertical lines on the middle and posterior edge. Background color of the dorsum is brown. A wide occipital band on the dorsum, formed by twelve transverse dark brown bars; some white scales on the posterior border of these bars. Dark brown lateral band with few yellowish scales dispersed into it, running from the shoulder to the groin; some white scales between the occipital and lateral bands; below the lateral band the flanks are yellowish. Limbs are brown with dark brown spots and some white scales dispersed. Tail is brown with some white scales dispersed and dark brown rings. Posterior third of the tail is immaculate brown. Ventrally, the throat, belly, limbs and tail are immaculate gray. Rear portion of belly and thighs are yellowish. Precloacal pores orange.

#### Variation.

There is no sexual dimorphism in size. In seven males: SVL: 57.4–69.9 mm. Axilla-groin distance: 21.4–28.7 mm. Head length: 15.1–17.2 mm. Head width: 11.2–13.0 mm. Head height: 6.4–8.9 mm. Foot length: 19.7–21.1 mm. Leg length: 37.1–46.2 mm. Arm length: 20.3–26.0 mm. Tail length: 101.6–111.3 mm (n = 2; autotomized in the rest). In three females: SVL: 57.3–65.6 mm. Axilla-groin distance: 25.6–32.8 mm. Head length: 15.3–15.8 mm. Head width: 11.1–12.1 mm. Head height: 6.2–6.7 mm. Foot length: 18.7–20.0 mm. Leg length: 37.2–39.0 mm. Arm length: 21.8–22.3 mm. Tail length 88.8–103.1 mm (n = 2; autotomized in the rest).

The variation of the scalation in *Liolaemus scorialis* is as follows. Midbody scales: 76–90 (x = 82.0 ± 4.7). Dorsal scales: 74–81 (x = 76.5 ± 4.3). Ventral scales 115–131 (x = 124.0 ± 6.0). Fourth finger lamellae: 21–24 (x = 22.7 ± 1.1). Fourth toe lamellae: 28–31 (x = 29.2 ± 1.4). Supralabial scales: 6–7 (x = 6.2 ± 0.4). Infralabial scales: 4–5 (x = 4.7 ± 0.5). Precloacal pores in males: 3–4. Interparietal scale pentagonal or hexagonal, bordered by 5–9 scales (x = 6.7 ± 1.2).

There is a slight sexual dichromatism, females have no yellowish color on the rear portion of belly and thighs. Males have the same color and pattern described for the holotype with variations only in shade. Females have the same color and pattern described for the holotype, but the background color of the dorsum can be brown or gray. One female lacks a wide occipital band because the transverse dark brown bars are not fused and it has an inconspicuous vertebral stripe. Also, in this female there are no lateral bands, since it has unfused vertical bars on the flanks. The tail has dark brown rings in both sexes. Males have orange precloacal pores. The coloration and pattern of the juveniles are unknown.

#### Distribution and natural history.

**Figure 4.**
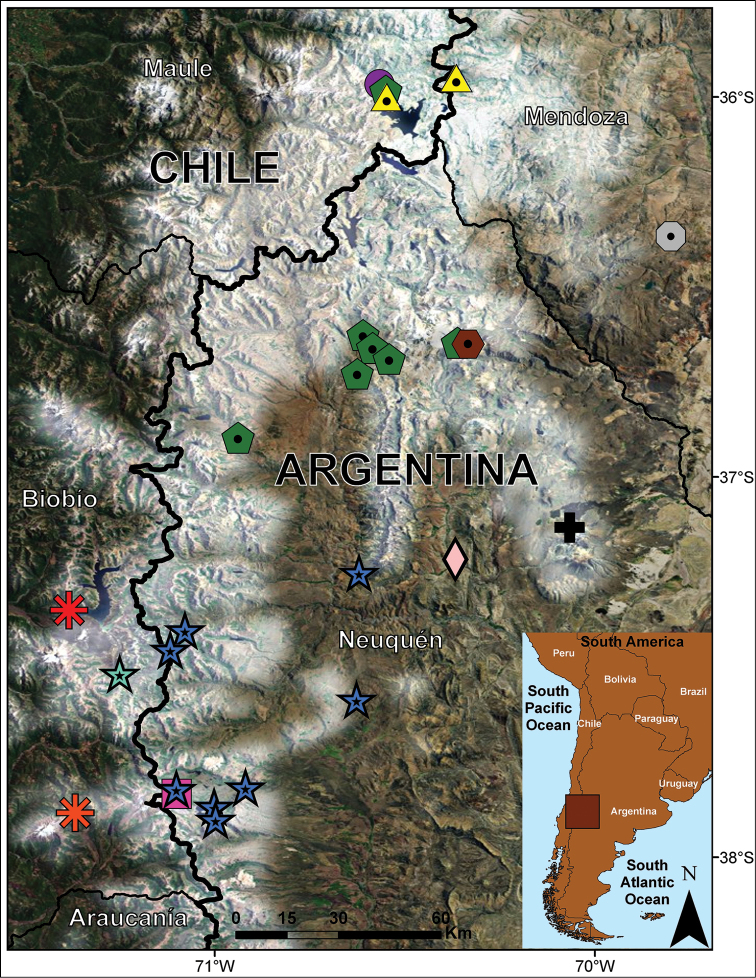
Distributional map for *Liolaemus scorialis* sp. n., *Liolaemus zabalai* sp. n. and the species of the *elongatus*-*kriegi* complex that inhabit in proximity of its. Asterisk: *Liolaemus scorialis* (red = near Laja Lagoon, type locality; orange = La Mula Lagoon). Star: *Liolaemus zabalai* sp. n. (light green = road to Los Barros, type locality; blue = distribution in Argentina). Purple circle: *Liolaemus carlosgarini*. Yellow triangle: *Liolaemus flavipiceus*. Green pentagon: *Liolaemus buergeri*. Gray octagon: *Liolaemus choique*. Brown hexagon: *Liolaemus antumalguen*. Black cross: *Liolaemus punmahuida*. Light pink diamond: *Liolaemus burmeisteri*. Pink square: *Liolaemus tregenzai*.

**Figure 5.**
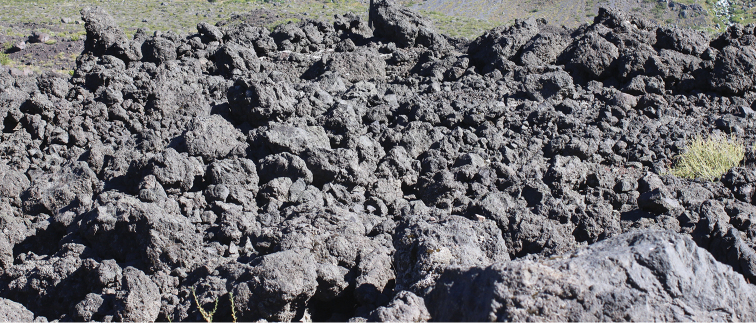
View of the type locality of *Liolaemus scorialis* sp. n., composed mainly of scoria volcanic rock.

The northern known distribution limit of the new species is the type locality, near the Laja Lagoon, 1450 m, Biobío Region, Chile (37°21'S – 71°23'W; Fig. 4). At the type locality, this new species was found inhabiting areas composed of sandy ground and volcanic sediments, where large accumulations of different sized igneous rocks protrude from the soil (Fig. 5). These sites correspond to a slag heap of solidified lava. The vegetational cover is low, consisting mainly of high-Andean forbs with species such as *Echium vulgare* and *Verbascum thapsus*, as well as the bush *Ephedra chilensis*. It is an abundant lizard of saxicolous habits. It was observed to be active between 9h00 and 18h00, taking refuge under the volcanic rocks. Also, we observed specimens in several places near the slopes of Antuco Volcano (37°23'S – 71°23'W, 1320 m; 37°23'S – 71°23'W, 1270 m; 37°23'S – 71°25'W, 1074 m) in similar environments. Near the Laja Lagoon, at its upper altitudinal limit (1450 m), this species was found in syntopy with *Phymaturus vociferator* Pincheira-Donoso, 2004. At 1320 m, it was found in syntopy with “*Liolaemus kriegi*/*Liolaemus* sp. A” and *Diplolaemus sexcinctus* Cei et al., 2003. At its lower altitudinal limit (1074 m), it was found in syntopy with *Liolaemus lemniscatus* Gravenhorst, 1838 and *Liolaemus tenuis* (Duméril & Bibron, 1837).

Its southern limit of distribution is in La Mula Lagoon (La Araucanía Region, Chile), 48 km South from the Antuco Volcano (37°53'S – 71°22'W), 1600 m. We have no data for vegetation or environment in La Mula Lagoon. In this location, according to the Herpetological Catalog of the Museo de Historia Natural of Concepción (unpublished), *Liolaemus scorialis* occurs in syntopy with *Liolaemus pictus* (Duméril & Bibron, 1837). However, this report probably actually refers to *Liolaemus septentrionalis* Pincheira-Donoso & Núñez, 2005 (fide Vera-Escalona et al. 2012). The Museo de Historia Natural of Concepción also listed an unidentified species of *Liolaemus* (labeled as *Liolaemus monticola* ssp., see discussion) and the snake *Tachymenis chilensis* Schlegel, 1837, from La Mula Lagoon.

he intestinal and stomach contents were examined: plant and insect remains were found in the intestine, along with a large number of nematodes of an unidentified species. No remains were found in the stomach. At the time of capture (January) two females had three embryos each and one female had several small oocytes.

### Liolaemus zabalai sp. n.

Taxon classificationAnimaliaSquamataLiolaemidae

http://zoobank.org/063D3CC3-0606-4CC4-8216-8F6B2B38CC3C

Fig. 6

Liolaemus kriegi , Donoso-Barros, 1974. Bol. Soc. Biol. Concepción, 47: 287.
Liolaemus kriegi (in part), Cei, 1986. Mus. Reg. Scien. Nat. Torino, 4: 230.
Liolaemus sp?, Torres-Pérez, 1997. Not. Biol., 5(4): 146.
Liolaemus kriegi (in part), Pincheira-Donoso, 2001. Not. Mens. Mus. Nac. Hist. Nat., Chile, 346: 11.
Liolaemus sp. A, Morando et al., 2003. Syst. Biol., 52: 179.
Liolaemus kriegi (in part), Pincheira-Donoso & Núñez, 2005. Pub. Oc. Mus. Nac. Hist. Nat., Chile, 59: 289.
Liolaemus kriegi (in part), Mella, 2005. Guía Camp. Rep. Chil. Zon. Cent., p. 64.
Liolaemus sp. A, Medina et al., 2013. Cuad. Herp. 27(1): 27.
Liolaemus sp. A, Medina et al., 2014. Biol. J. Linnean Soc. 113: 256.

#### Holotype.

**Figure 6.**
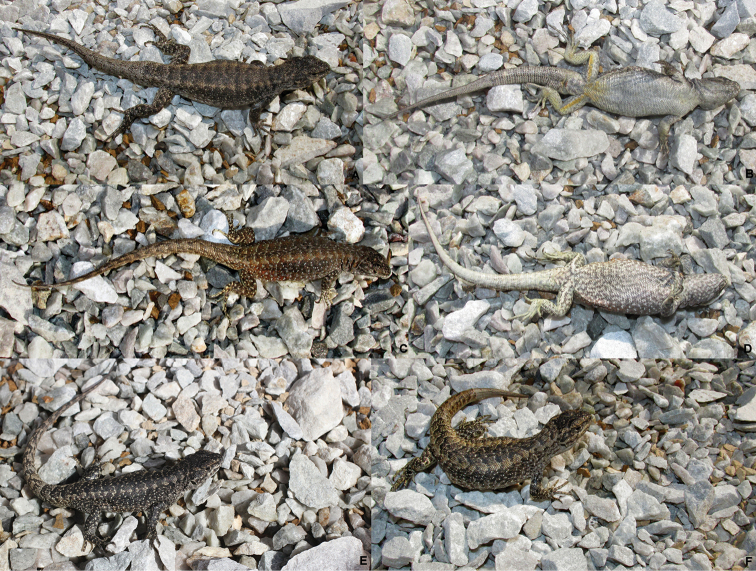
*Liolaemus zabalai* sp. n. **A, B** Holotype, male **C, D** Paratype, female **E** Paratype, male **F** Paratype, female. All from the type locality, near Los Barros, Laja Lagoon, Biobío Region, Chile.

SSUC Re 602 (Fig. 6). Near Los Barros, Laja Lagoon, Biobío Region, Chile. (37°31'S – 71°15'W, 1460 m). Collected by J. Troncoso-Palacios, F. Urra and H. Díaz. 07/01/2014.

#### Paratypes.

SSUC Re 598. Adult male. SSUC Re 597, 599, 600–01. Four adult females. The same data as the holotype (Figs 6 and 8). MZUC 35607, 39567. One male and one female. Malleco, Antuco Volcano, Los Barros. Unknown coll.

#### Etymology.

This species is named after Patricio Zabala, collection manager of the “Colección de Flora y Fauna Patricio Sánchez Reyes, Pontificia Universidad Católica de Chile” (SSUC). We dedicate this species to him because of his support of herpetological research in Chile, allowing us to review and deposit material in SSUC, and especially for his friendship.

#### Diagnosis.

**Figure 7.**
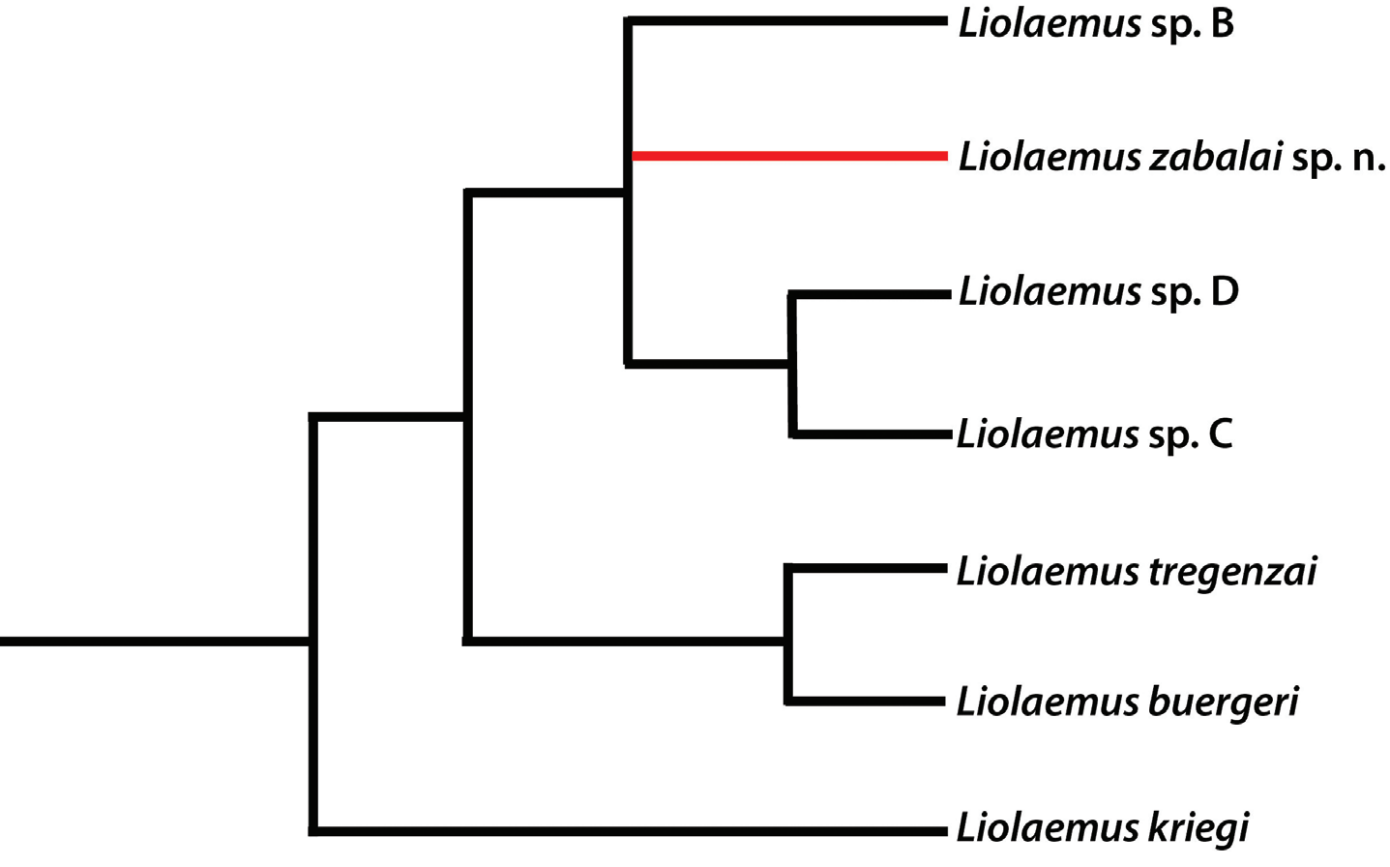
Phylogenetic position of *Liolaemus zabalai* sp. n. in the *kriegi* clade, based on cytochrome-b (cyt-b) locus according to Medina et al. (2014).

*Liolaemus zabalai* belongs to the *kriegi* clade of the *elongatus*-*kriegi* complex and is closely related to some undescribed species: *Liolaemus* sp. C and *Liolaemus* sp. D; being more distant from the currently described species *Liolaemus buergeri*, *Liolaemus kriegi* and *Liolaemus tregenzai* (Fig. 7). According to Medina et al. (2014), in regards to the species of the *kriegi* clade *Liolaemus zabalai* is sympatric only with *Liolaemus tregenzai* at the Copahue Volcano.

With respect to the species species of the *kriegi* clade, *Liolaemus zabalai* differs from *Liolaemus tregenzai* because the latter has 71–85 midbody scales and the males have no precloacal pores (Pincheira-Donoso and Scolaro 2007), whereas *Liolaemus zabalai* has 90–104 midbody scales and the males have 3–5 precloacal pores. In addition, the green-bluish ventral color of *Liolaemus tregenzai* is completely absent in *Liolaemus zabalai*. The uncorrected pairwise difference (cyt-b) between the species is 3.09% (Medina et al. 2014).

*Liolaemus zabalai* differs from *Liolaemus kriegi* in that the latter reaches 101.1 mm SVL, has reddish cloacal coloration in both sexes and has an unringed tail (Avila et al. 2003), whereas *Liolaemus zabalai* is smaller (max. SVL = 92.0 mm), has yellowish cloacal coloration in both sexes and has a ringed tail (in specimens with original tails). The uncorrected pairwise difference between these species is 3.79% (Medina et al. 2014).

**Figure 8.**
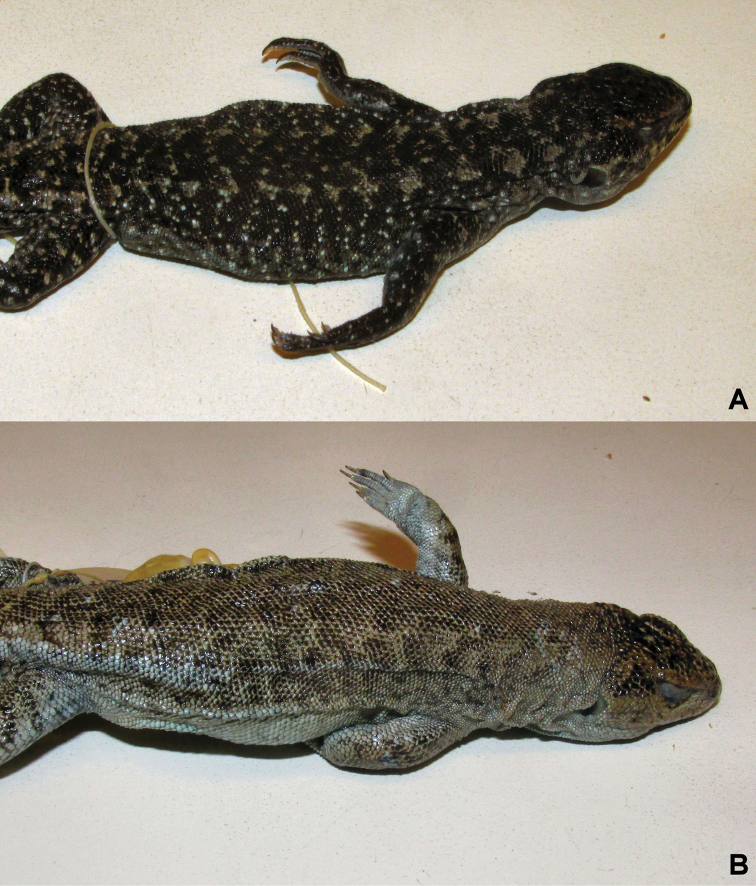
Comparison of the dorsal color pattern. **A** *Liolaemus zabalai* sp. n. with marked color pattern and **B** *Liolaemus buergeri*, diffuse color pattern.

*Liolaemus zabalai* differs from *Liolaemus buergeri* in that the latter has fewer dorsal scales (78–91; x = 84.1 ± 4.4, n = 14) than *Liolaemus zabalai* (86–96; x = 89.4 ± 3.2, n = 8) (Mann–Whitney U = 19.5; P = 0.01, DF = 20). *Liolaemus zabalai* has more loreal scales between the nasal and the subocular (4–6; x = 4.3 ± 0.6, n = 8) than *Liolaemus buergeri* (3–4; x = 3.3 ± 0.5, n = 14) (Mann–Whitney U = 11.0; P < 0.01, DF = 20). Also, *Liolaemus buergeri* has a vertebral stripe on the tail, whereas *Liolaemus zabalai* has a ringed original tail. The limbs in *Liolaemus zabalai* are black with dispersed light brown spots, whereas *Liolaemus buergeri* has brown limbs with dispersed black spots (Fig. 8). *Liolaemus zabalai* and *Liolaemus buergeri* share basically the same dorsal coloration pattern, but this is noticeably more marked and darker in *Liolaemus zabalai* (Fig. 8, see discussion). Based on the cyt-b locus, the uncorrected average pairwise difference between *Liolaemus zabalai* and *Liolaemus buergeri* is 2.94% (Medina et al. 2014), greater than the values reported for other *Liolaemus* widely accepted as valid species (see discussion). Also, *Liolaemus zabalai* can vocalize, a feature only documented for *Liolaemus chiliensis* in the entire genus *Liolaemus* (Labra et al. 2013). Finally, although the ranges overlap, males of *Liolaemus buergeri* have 3–4 (x = 3.3) precloacal pores, whereas males of *Liolaemus zabalai* have 3–5 (x = 3.9) precloacal pores (Medina et al. 2014).

Compared to the other species of the *elongatus*-*kriegi* complex that occur near the known distribution of *Liolaemus zabalai*, the new species may be diagnosed as follows. Males of *Liolaemus zabalai* have precloacal pores, whereas males of *Liolaemus flavipiceus* and *Liolaemus punmahuida* lack them (Table 3). *Liolaemus zabalai* is larger than *Liolaemus scorialis*; and *Liolaemus zabalai* has more midbody scales than *Liolaemus antumalguen*, *Liolaemus burmeisteri* and *Liolaemus choique* (Table 3).

#### Description of the holotype.

Adult male. SVL: 90.3 mm. Tail length: 92.3 mm (autotomized). Axilla-groin length 39.7 mm. Head length (from the posterior border of the auditory meatus to the tip of the snout) 22.2 mm. Head width (distance between the two ear openings) 16.5 mm. Head height (at the level of ear openings) 11.7 mm. Forelimb length 28.5 mm. Hindlimb length 47.1 mm. Foot length 23.4 mm. Rostral scale wider (4.5 mm) than high (2.2 mm). Two postrostrals. Four internasals. Heptagonal interparietal scale, with a central, small, and whitish central spot marking the position of the parietal eye. Interparietal smaller than right parietal, but bigger than left parietal, surrounded by eight scales: nine scales between the interparietal and the rostral; 14 scales between occiput and rostral; orbital semicircle complete on both sides (formed by 13 scales); 5 supraoculars on both sides; seven superciliary scales. Frontal area is divided into six scales (three posterior, one anterior-left, two anterior-rigth); 2 scales between nasal and canthal; preocular separated from the lorilabials by one loreal scale; nasal in contact with the rostral, surrounded by six scales. There is one row of lorilabials between the supralabials and the subocular. Seven supralabials, the fourth is curved upward without contacting the subocular. Five infralabial scales. The mental scale is pentagonal and is in contact with four scales. Four pairs of postmental shields, the second is separated by two scales. Temporal scales are subimbricated and smooth or slightly keeled. Nine temporal scales between the level of superciliary scales and the rictal level. Two projected scales on the anterior edge of the ear, which are small and do not cover the auditory meatus. There is no differentiated auricular scale. Forty-two gulars between auditory meatus. Well developed “Y” shaped lateral neck fold with antehumeral and posthumeral folds developed. Dorsolateral fold slightly developed. Midbody scales 90. Dorsal scales on the vertebral zone are lanceolate to rounded, subimbricate, keeled and without mucrons. Dorsal scales on the paravertebral fields are more rounded, subimbricate, smooth or with less developed keels, without mucrons and there are interstitial granules between them. Dorsal scales are smaller than the ventral scales. Dorsal scales 86. Ventral scales are rhomboidal, smooth, subimbricate, and with few interstitial granules. Ventral scales 122. There are three precloacal pores. The suprafemoral scales are rhomboidal, imbricate, and smooth or keeled. Infrafemoral scales are lanceolate to rhomboidal, smooth, and subimbricate and with few interstitial granules. Supra-antebrachials scales are rhomboidal to rounded, subimbricate, and keeled or smooth. Infra-antebrachials are rounded to rhomboidal, subimbricate, and smooth. The dorsal scales of the tail are lanceolate to rectangular, subimbricate, keeled or smooth and with few interstitial granules. The ventral scales of the tail vary from lanceolate to triangular, and are subimbricate and smooth. Lamellae of the fingers: I: 11, II: 16, III: 20, IV: 22 and V: 15. Lamellae of the toes: I: 12, II: 16, III: 21, VI: 27 and V: 18.

#### Color of the holotype in life.

Black head, with some light brown spots on the supraocular and snout areas. The scales located behind the orbital semicircles are light brown; but the interparietal scale, parietal scales and the scales in contact with the parietal scales are black. Superciliary scales are light brown with black spots. Temporal scales are light brown; cheeks light gray with some black spots. Subocular is gray with a black vertical line on the middle. Background color of the dorsum is light brown. Wide occipital band on the dorsum, formed by twelve transverse black bars. Very few whitish scales dispersed on the dorsum. Black lateral band bearing a few dispersed whitish scales, running from the tip of snout to the groin. Flanks below lateral band are light brown. Limbs black with dispersed light brown spots. Tail light brown with inconspicuous vertebral stripe in the regenerated zone; occipital black band ends in the first fifth of the tail, remainder with some dispersed black spots and a black vertebral stripe. Throat, belly and ventral surfaces of limbs whitish with dispersed inconspicuous dark dots. Rear portion of the belly and the thighs are yellowish. Ventrally, tail is whitish with a dark gray ventral stripe and diffuse dark gray rings from the cloaca to the midpoint of the tail. Precloacal pores orange.

#### Variation.

In three males: SVL: 72.6–90.3 mm. Axilla-groin distance: 32.7–38.6 mm. Head length: 17.6–22.2 mm. Head width: 14.2–16.5 mm. Head height: 9.2–11.7 mm. Foot length: 21.5–23.0 mm. Leg length: 42.1–47.2 mm. Arm length: 24.6–28.5 mm. Tail length: 102.0 mm in one specimen (autotomized in the rest). In three females: SVL: 71.8–90.2 mm. Axilla-groin distance: 32.9–42.7 mm. Head length: 17.9–19.5 mm. Head width: 13.9–16.6 mm. Head height: 9.4–11.1 mm. Foot length: 20.6–24.2 mm. Leg length: 41.5–48.8 mm. Arm length: 24.8–29.4 mm. Tail length: 105–115 mm (in two specimens without autotomized tails).

The variation of the scalation in *Liolaemus zabalai* is as follows. Midbody scales: 90–104 (x = 94.3 ± 4.8). Dorsal scales: 86–96 (x = 89.4 ± 3.2). Ventral scales 116–122 (x = 119.5 ± 2.1). Fourth finger lamellae: 19–22 (x = 20.9 ± 1.0). Fourth toe lamellae: 26–27 (x = 26.8 ± 0.5). Supralabial scales: 6–7 (x = 6.6, ± 0.5). Infralabial scales: 4–5 (x = 4.6 ± 0.5). Interparietal scale pentagonal, hexagonal or heptagonal, bordered by 5–8 scales (x = 7.3 ± 1.1). Precloacal pores in males: 3–4.

There is slight sexual dichromatism; males are slightly darker than females. In general, all specimens have the pattern and color described for the holotype. One female has rusty-colored scales dispersed on the flanks, paravertebral fields and groin. In all specimens, the ventral surface of the throat, belly and limbs are whitish with dark marked or inconspicuous dots dispersed; there is a fragmented midventral stripe on the belly of two specimens. Males and females have a yellowish coloration in the posterior portion of the belly and the thighs (faint in some females). The tail has black rings, marked or diffuse, with a fragmented vertebral stripe in all specimens with complete original tails. Males have orange precloacal pores. The coloration and pattern of the juveniles are unknown.

#### Distribution and natural history.

**Figure 9.**
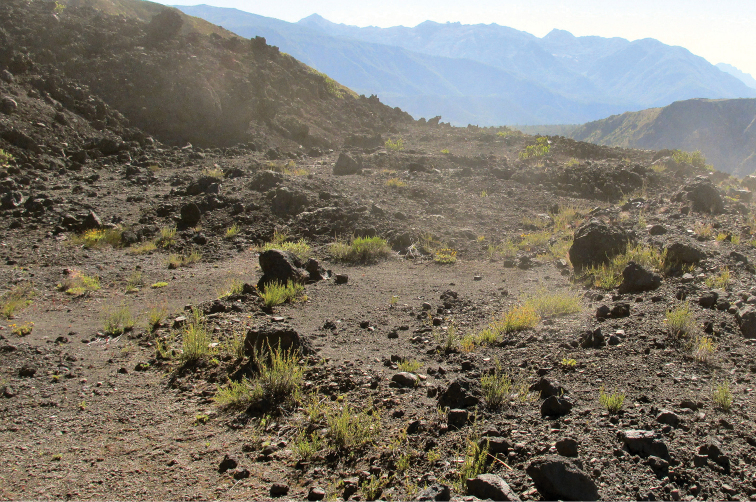
View of the type locality of *Liolaemus zabalai* sp. n.

To our knowledge, in Chile this species is only found in the surroundings of the Laja Lagoon. The type locality is near Los Barros, Laja Lagoon, Biobío Region, Chile (37°31'S – 71°15'W, 1460 m, Fig. 9); but we also saw specimens (not collected) on the road to the Laja Lagoon at two localities (37°23'S – 71°23'W, 1320 m; 37°23'S – 71°22'W, 1390 m). The new species was found inhabiting areas of sandy soil with rocks of small and medium size. The vegetational cover is low, consisting mainly of *Ephedra chilensis*. It is an abundant lizard of saxicolous habits. This species was observed active between 11h00 and 18h00, taking refuge in cavities under the rocks. Near Los Barros, at its upper altitudinal limit (1460 m), this species was found in syntopy with *Diplolaemus sexcinctus*. At the lower altitudinal limit (1320 m), it was found in syntopy with *Liolaemus scorialis*, *Phymaturus vociferator* and *Diplolaemus sexcinctus*. Two specimens of *Liolaemus zabalai* vocalized (squealed) in several occasions in response to the manipulation.

*Liolaemus zabalai* is also found in Argentina (where it has been called “*Liolaemus* sp. A”) at several localities in Neuquén Province (Morando et al. 2003, Medina et al. 2013, 2014).

An analysis of the intestinal contents performed on one specimen, showed that this species is omnivorous, but feeds mainly on plants. At the time of capture (January) the females had no embryos, but three had several small oocytes.

## Discussion

In this work, the taxonomic status of two Chilean populations of the *Liolaemus elongatus*-*kriegi* complex from the Laja Lagoon have been clarified, here newly described as *Liolaemus zabalai* (previously confused with *Liolaemus kriegi* and also designed as *Liolaemus* sp. A) and *Liolaemus scorialis*. Pincheira-Donoso (2001) recorded two species of the *Liolaemus elongatus*-*kriegi* complex from the same location: *Liolaemus kriegi* and *Liolaemus buergeri*. Even though we did not examine the three specimens of “*Liolaemus buergeri*” listed by Pincheira-Donoso (2001), we believe that these correspond to *Liolaemus scorialis*, since the aspect of this new species resembles *Liolaemus buergeri* (although it is notably smaller than it) and we did not find additional species of the *elongatus*-*kriegi* in the vicinity of Laja Lagoon. Also, Troncoso-Palacios et al. (2012) published several photographs of specimens from a population of “*Liolaemus buergeri*” from Los Humos, Libertador Bernardo O`Higgins Region, Chile, but unfortunately those specimens were not collected. This population is completely isolated from other populations of *Liolaemus buergeri* and some specimens exhibit a completely black ventral coloration, a feature absent in other populations of *Liolaemus buergeri* (Donoso-Barros 1966, Pincheira-Donoso and Núñez 2005). A more conclusive study in regard to this population should be conducted. Besides, there is diverse evidence supporting the existence of at least three more undescribed species currently assigned to *Liolaemus buergeri* in Argentina (Medina et al. 2013, 2014, Morando et al. 2003).

Assigning *Liolaemus scorialis* to any of the groups (Lobo 2005, Lobo et al. 2010b) or clades (Morando et al. 2003, Avila et al. 2012) proposed for such a diverse lineage of Patagonian lizards is a difficult task, especially taking into account that the phylogenetic studies based on morphological and molecular data disagree, and unfortunately we do not have molecular data for *Liolaemus scorialis*. However, it is unlikely that *Liolaemus scorialis* belongs to the *leopardinus* group-clade, because it completely lacks “leopard-like” dorsal spots, a distinctive feature of these lizards (Lobo 2005). Also, it is unlikely that *Liolaemus scorialis* belongs to the *capillitas* group, because species of this group share two synapomorphies absent in *Liolaemus scorialis*: spots in the shoulder region and a red coloration in the cloacal zone (Abdala et al. 2010, Lobo 2005). The *petrophilus* clade (Avila et al. 2012, Morando et al. 2003) includes all species of the *capillitas* group (with the exception of *Liolaemus heliodermis*, not sampled) plus *Liolaemus austromendocinus*, *Liolaemus gununakuna*, *Liolaemus parvus* and *Liolaemus petrophilus*. However, with the exception of *Liolaemus petrophilus* and *Liolaemus gununakuna*, all species of the *petrophilus* clade have fewer than 82 midbody scales (Abdala et al. 2010, Avila et al. 2004, Espinoza and Lobo 2003, Quinteros et al. 2008), whereas *Liolaemus scorialis* has 76–90 midbody scales. In regards to the *punmahuida* clade (Avila et al. 2010a), included into the *elongatus* group by Lobo et al. (2010b), both species of this clade (*Liolaemus flavipiceus* and *Liolaemus punmahuida*) have red coloration in the cloacal zone and males lack precloacal pores (Avila et al. 2003, Cei and Videla 2003), features absent in *Liolaemus scorialis*. *Liolaemus scorialis* is probably related to the *elongatus* or *kriegi* clades, as some species of these clades occur in the vicinity or in the type locality of *Liolaemus scorialis* and have similar counts of midbody, dorsal and ventral scales. Also, some of these species have white dorsal dots, rings on the tail and yellow in the cloacal zone (Abdala et al. 2010, Avila et al. 2010a, 2012, Cei 1986) like *Liolaemus scorialis*. A molecular phylogeny including *Liolaemus scorialis* is required to clarify this.

In the case of *Liolaemus zabalai* of the *kriegi* clade, the uncorrected pairwise differences between it and other species of the *kriegi* clade are 2.94–3.79%, almost at the limit of the value (3%) proposed for identify candidate species in *Liolaemus* (Breitman et al. 2012). In comparison, other *Liolaemus* lizards widely accepted as valid species show a lower level of differentiation for the mitochondrial gene cyt-b, for example: *Liolaemus martorii* Abdala, 2003 vs. *Liolaemus morenoi* Etheridge & Christie, 2003, 2.73% (Avila et al. 2010b); *Liolaemus riojanus* Cei, 1979 vs. *Liolaemus multimaculatus* (Duméril & Bibron, 1837), 1.23% (Avila et al. 2009); *Liolaemus chacabucoense* Núñez & Scolaro, 2009 vs. *Liolaemus kingii* (Bell, 1843), 2.22% (Breitman 2013). *Liolaemus zabalai* can vocalize, a trait only documented for *Liolaemus chiliensis* (Labra et al. 2013) and also taken as diagnostic feature in *Liolaemus* (Pincheira-Donoso and Núñez 2005: 232) and the closely related genus *Phymaturus* (Lobo et al. 2010a: 118). Regarding the morphological diagnosis included in previous studies, Pincheira-Donoso and Núñez (2005) reviewed two specimens of *Liolaemus kriegi* from Laja Lagoon (here described as *Liolaemus zabalai*), which they described and provided the following diagnosis “the species is very similar to *Liolaemus buergeri*, differing in that the latter has a lighter color, brown or dark brown; in combination with a smaller number of keeled scales on the dorsum” (Pincheira-Donoso and Núñez 2005: 289, our translation). Here, we find the same color difference, and expand the differences in scalation; although we found no differences in the number of dorsal scales. Medina et al. (2013) recorded a similar maximum SVL (86.3 mm) compared to us (92.0 mm). Also, Medina et al. (2013) based on a discriminant analysis of several continuous and meristic characters, reported that *Liolaemus zabalai* (designated as “*Liolaemus* sp. A” in its study) has sexual dimorphism, with a sample of 21 females and 23 males. We were unable to replicate the statistical analysis to confirm this sexual dimorphism because our sample is small (5 females and 3 males). Also, Medina et al. (2013) recorded 3–5 precloacal pores in the males (n = 23), whereas we recorded only 3–4 (n = 3). Eventhough we found *Liolaemus scorialis* and *Liolaemus zabalai* in syntopy, *Liolaemus scorialis* was found mainly in a solid lava slag heap (where it was the only species recorded in this environment), whereas *Liolaemus zabalai* was found in bushy-rocky environments together with specimens of *Liolaemus scorialis* and other lizards. Regarding the population of “*Liolaemus kriegi*” from Cordillera de Curicó in Chile, 35°10'S (Donoso-Barros 1966), we have doubts about its real identity, especially considering that according to Medina et al. (2014) *Liolaemus kriegi* is distributed south of 38°40'S latitude (coordinates transformed by us).

Torres-Pérez (1997) recorded two *Liolaemus* sp. from Laja Lagoon. He pointed that one of them has 92 midbody scales, brown color and precloacal pores in males. It is difficult to try an identification, but the midbody scale count match with *Liolaemus zabalai*. Torres-Pérez (1997) indicated that the other *Liolaemus* sp. has no precloacal pores. It match only with *Liolaemus chillanensis* Müller & Hellmich, 1932, recorded in the Laja Laagon (Pincheira-Donoso and Núñez 2005).

In this study, *Liolaemus ceii* is considered a junior synonym of *Liolaemus kriegi*. This synonymy was recommended by Morando et al. (2003) because they did not find genetic evidence to differentiate both species. Recently, Medina et al. (2014) performed a wider genetic study and found that these two species form one lineage, called “*Liolaemus kriegi* + *ceii*”. Because individuals from both type localities show some morphological differences, they proposed two hypothesis: (1) *Liolaemus ceii* and *Liolaemus kriegi* constitute two species, for which different environments prompted relatively rapid and recent morphological divergence with insufficient time for molecular differentiation; and (2) they are conspecific and show clinal morphological variation owing to local adaptations (Medina et al. 2014). However, the published literature regarding *Liolaemus ceii* and *Liolaemus kriegi* (Cei 1986, Donoso-Barros 1971) does not include enough morphological comparison between them. We believe that for the moment *Liolaemus ceii* should be considered as a junior synonym of *Liolaemus kriegi*, because published morphological evidence to support *Liolaemus ceii* as full species is insufficient and the results of genetic studies (Medina et al. 2014, Morando et al. 2003) do not support to *Liolaemus ceii* as full species.

*Liolaemus chillanensis* was included in the *elongatus* clade by Avila et al. (2010a) and Avila et al. (2012) based on mitochondrial DNA data generated by Torres-Pérez et al. (2009), but at least part of the specimens used as vouchers were misidentified (Troncoso-Palacios, unpublished data). Therefore, in this study we do not consider *Liolaemus chillanensis* as a member of the *elongatus*-*kriegi* complex and we excluded it from our comparisons. Also, we examined one male of *Liolaemus monticola* ssp. (MRC 676) syntopic with *Liolaemus scorialis* in La Mula Lagoon, and identified it as *Liolaemus neuquensis* Müller & Hellmich, 1939, a species described from Copahue Volcano (Müller and Hellmich 1939b), 15 km E from La Mula Lagoon; being the first record of *Liolaemus neuquensis* in Chile.

In summary, this work describes two new species of the *elongatus*-*kriegi* complex lizards from the vicinity of the Laja Lagoon, in southern Chile, one probably confused with *Liolaemus buergeri*: *Liolaemus scorialis* and the other with a history of mis-identifications as *Liolaemus kriegi* or *Liolaemus* sp. A, for which we provide the formal name *Liolaemus zabalai*. Nonetheless, there is certainly still much to discover about the diversity of this group of Patagonian lizards.

## Supplementary Material

XML Treatment for Liolaemus scorialis

XML Treatment for Liolaemus zabalai

## Appendix 1

**Specimens examined.** Museum codes are as follow: LCUC (Laboratorio de Citogenética, Facultad de Ciencias, Universidad de Chile), MNHN-CL (Museo Nacional de Historia Natural, Chile), MRC (Museo de Historia Natural de Concepción), MZUC (Museo de Zoología de la Universidad de Concepción) and SSUC (Colección de Flora y Fauna Patricio Sánchez Reyes, Pontificia Universidad Católica de Chile).

*Liolaemus buergeri*. LCUC 2311. El Planchón, 2370 m. M. Lamborot & M.E. Manzur colls. 07/01/1996. SSUC Re 434–37. El Planchón, road to Teno Lagoon. J. Troncoso-Palacios, L. Negrete & R. Barros colls. January, 2012. SSUC Re 171–180. Maule Lagoon. F. Ferri coll. 20/02/2011.

*Liolaemus carlosgarini*. MNHN-CL 4531–67. Road to Maule Lagoon. C. Garín coll. 22/02/2008. SSUC Re 181–189, 349. Road to Maule Lagoon. F. Ferri coll. 20/02/2011.

*Liolaemus cristiani*. SSUC Re 537. El Peine. J. Troncoso-Palacios coll. 28/11/2011.

*Liolaemus flavipiceus*. MNHN-CL 2118, 2120. Maule Lagoon. C. Veloso & S. Silva colls. MNHN-CL 2167, 2170. Maule Lagoon. J.C. Torres-Mura & H. Núñez. MNHN-CL 4399–07. Laguna del Maule, aguas abajo, 2153 m. C. Garín & G. Lobos colls. 03/03/2008. SSUC Re 169–70. Maule Lagoon. F. Ferri coll. 20/02/2011.

*Liolaemus frassinettii*. LCUC 800–01. Cantillana. Unknown coll. 14/04/1983. SSUC Re 80. Altos de Cantillana. F. Torres coll.

*Liolaemus leopardinus*. MNHN-CL 3437–3439. El Colorado. H. Núñez, C. Garín, V. Meriggio, S. Fox & S. Perea colls. 06/01/2001. MNHN-CL 4025, 4027–28. Farellones. C. Veloso coll. 11/01/1988. MNHN-CL 4890–91. El Colorado. D. Esquerré, M. Palma, S. Fox & E. Santoyo colls. February, 2012. SSUC Re 364. Farellones. F. Ferri coll. 12/10/2010. SSUC Re 365. Farellones. F. Ferri coll. 13/02/2011. SSUC Re 366–67. Farellones. F. Ferri, M.L. Carrevedo & J. Troncoso-Palacios colls. 25/01/2012.

*Liolaemus neuquensis*. MRC 676. La Mula Lagoon, Araucanía Region, Chile. Unknown coll.

*Liolaemus ramonensis*. MNHN-CL 4007–08, 4012, 4015–17. Quebrada de Macul. C. Veloso & P. Espejo colls. 06/03/1987.

*Liolaemus scorialis*. SSUC Re SSUC Re 612-17. 7 km NW of the summit of the Antuco Volcano, near the Laja Lagoon, Biobío Region, Chile. J. Troncoso-Palacios, F. Urra & H. Díaz colls. 08/01/2014. MRC 675, 677, 680, 682. La Mula Lagoon, Ralco National Reserve. Unknown coll. 01/12/2001.

*Liolaemus ubaghsi*. MNHN-CL 3808–16. Chapa Verde. H. Núñez, C. Garín & D. Pincheira-Donoso colls. 22–23/05/2003. MNHN-CL 1601. Chapa Verde. M. Elgueta coll. SSUC Re 491–92. Tranque Barahona, O’Higgins Region, Chile. R. Thomsom & G. Ugalde colls. 15/04/2008.

*Liolaemus valdesianus*. SSUC Re 129. Cajón del Maipo. Unknown coll. SSUC Re 363. Lo Valdés. F. Ferri coll. 10/01/2011. SSUC Re 559. El Yeso. C. Garín coll. 20/02/2013.

*Liolaemus zabalai*. SSUC Re 597–602. Near Los Barros, Laja Lagoon, Biobío Region, Chile. Collected by J. Troncoso-Palacios, F. Urra and H. Díaz. 07/01/2014. MZUC 35607, 39567. Malleco, Volcán Antuco, Los Barros. Unknown coll.
